# Quantifying the relationship between within-host dynamics and transmission for viral diseases of livestock

**DOI:** 10.1098/rsif.2023.0445

**Published:** 2024-02-14

**Authors:** Simon Gubbins

**Affiliations:** The Pirbright Institute, Ash Road, Pirbright, Surrey GU24 0NF, UK

**Keywords:** mathematical modelling, Bayesian methods, foot-and-mouth disease, swine influenza, cattle, pigs

## Abstract

Understanding the population dynamics of an infectious disease requires linking within-host dynamics and between-host transmission in a quantitative manner, but this is seldom done in practice. Here a simple phenomenological model for viral dynamics within a host is linked to between-host transmission by assuming that the probability of transmission is related to log viral titre. Data from transmission experiments for two viral diseases of livestock, foot-and-mouth disease virus in cattle and swine influenza virus in pigs, are used to parametrize the model and, importantly, test the underlying assumptions. The model allows the relationship between within-host parameters and transmission to be determined explicitly through their influence on the reproduction number and generation time. Furthermore, these critical within-host parameters (time and level of peak titre, viral growth and clearance rates) can be computed from more complex within-host models, raising the possibility of assessing the impact of within-host processes on between-host transmission in a more detailed quantitative manner.

## 1. Introduction

A pathogen must replicate to a sufficiently high level within an infected host for it to be able to sustain ongoing chains of transmission to new hosts. Consequently, linking within-host dynamics and between-host transmission in a quantitative and predictive manner is important for understanding the population dynamics of an infectious disease, yet it is seldom done in practice [1–4]. Furthermore, most studies which have considered the link between pathogen load and transmission have relied on plausible assumptions rather than empirical data [1,2].

The dose–response relationship between viral load and transmission has been estimated for viruses such as HIV-1 in humans [5,6], dengue virus in humans and mosquitoes [7], herpes simplex virus-2 in humans [8] and swine influenza virus (SwIV) in pigs [9]. More recently, studies have used models to describe viral dynamics within a host and linked these to transmission using dose–response models, for example, for foot-and-mouth disease virus (FMDV) in cattle [10], SARS-CoV-2 in humans [11,12], Zika virus in humans and mosquitoes [13] and Rift Valley fever virus in cattle, goats, sheep and mosquitoes [14].

Although these approaches have linked within-host dynamics and transmission, they did not explicitly explore how processes at one scale (i.e. within-host dynamics) influence those at another (i.e. between-host transmission) in detail (cf. [15,16]). For example, they did not quantify how viral growth or clearance rates impact the reproduction number (i.e. the expected number of secondary infections arising from the individual) or the generation time (i.e. the interval between an individual becoming infected and it infecting others).

In this paper, a simple phenomenological model for within-host viral dynamics [10,17,18] was linked to between-host transmission by assuming that the probability of transmission is related to viral titre [2]. This model was then used to derive expressions relating the within-host and transmission parameters to the reproduction number and the generation time. The model was parametrized and assumptions tested using data from a series of one-to-one transmission experiments for two viruses that infect livestock: FMDV in cattle [19] and SwIV in pigs [9]. Importantly, these experiments used viruses in their natural hosts and animals that were infected by a natural route (contact with infected animals), rather than inoculation, thereby making them less artificial and more reflective of a real-world situation. Furthermore, the data allowed heterogeneity among animals in within-host dynamics and transmission to be explored.

## 2. Methods

### 2.1. Data

**Figure 1.**
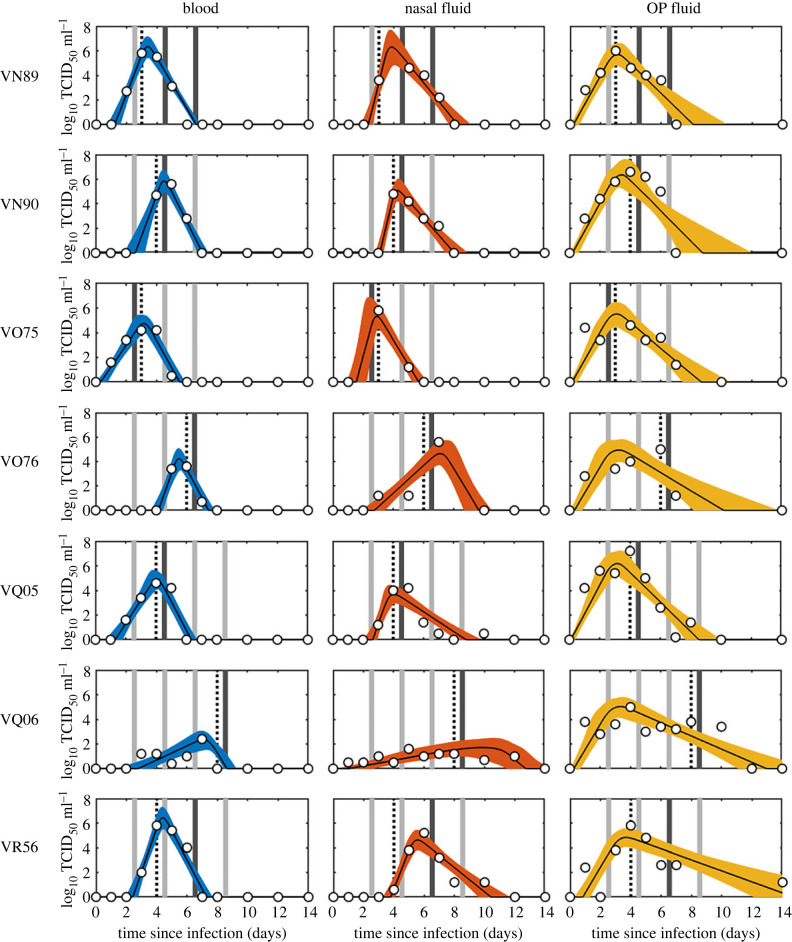
Within-host viral dynamics and transmission of foot-and-mouth disease virus in cattle. Each plot shows the viral titre (log_10_ tissue culture (TC) ID_50_ ml^−1^) in blood (blue), nasal fluid (orange) or oesophageal–pharyngeal (OP) fluid (yellow) for the animal (identified to the left of the first column): posterior median (solid black line) and 2.5th and 97.5th percentiles (shading) for the fitted virus curves given by equation (2.1). Open circles give the observed viral titres for each animal. Timing and outcome of experimental challenges are indicated by bars, which are coloured dark grey if the challenge was successful and light grey if it was not. The vertical black dashed lines indicate the onset of clinical signs.

**Figure 2.**
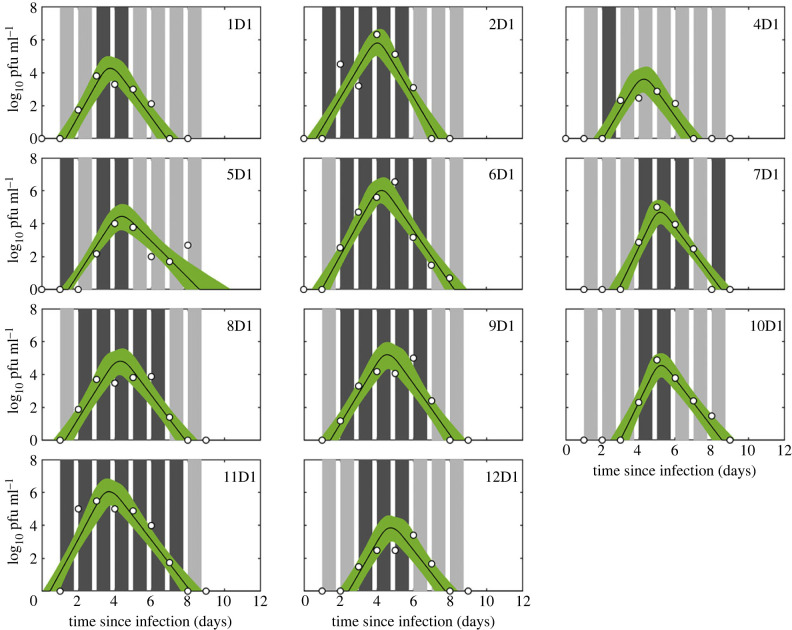
Within-host viral dynamics and transmission of swine influenza virus in pigs. Each plot shows the viral titre (log_10_ pfu ml^−1^) in nasal swabs for the animal (identified in the top right-hand corner): posterior median (solid black line) and 2.5th and 97.5th percentiles (green shading) for the fitted virus curves given by equation (2.1). Open circles give the observed viral titres for each animal. Timing and outcome of experimental challenges are indicated by bars, which are coloured dark grey if the challenge was successful and light grey if it was not.

The relationship between within-host dynamics and transmission was investigated using previously published data from a series of one-to-one transmission experiments for FMDV (O UKG 34/2001) in cattle [19] and SwIV (H1N1pdm09) in pigs [9]. In both cases, uninfected recipient animals were challenged by exposure to infected donor animals at multiple times post infection of the donor and the outcome of challenge recorded (i.e. whether or not transmission occurred; figures 1 and 2). The donor animals were infected by direct contact challenge with another infected animal, rather than by inoculation. As well as challenge outcome, viral titres were measured daily in the donor animals. In the FMDV experiments, titres were measured in three compartments: blood, nasal fluid (NF) and oesophageal–pharyngeal fluid (OPF) (figure 1). In the SwIV experiments, titres were measured daily in nasal swabs (figure 2). All data are available from the original publications [9,19], but are provided in the electronic supplementary material (data S1 and S2) in the format used in the present analysis.

### 2.2. Within-host viral dynamics

Within-host viral titres typically rise exponentially following infection, reaching a maximum level after which they decay exponentially. This pattern can be captured using a simple phenomenological model [10,17,18] in which the viral titre at *τ* days post infection is given by

2.1 $$V(\tau )=\frac{2V_{p}}{\exp(-\lambda_{g}(\tau -T_{p}))+\exp(\lambda_{d}(\tau -T_{p}))},$$

where *V_p_*, *T_p_*, *λ_g_* and *λ_d_* are the peak titre, the time of peak titre and the rates during the exponential growth and decay phases, respectively.

Individual variation in the within-host viral dynamics described by equation (2.1) was incorporated by allowing each of the parameters (i.e. *V_p_*, *T_p_*, *λ_g_* and *λ_d_*) to differ among individuals. Specifically, the parameters were assumed to be drawn from higher-order distributions, so that

2.2 $$\begin{array}{rl} \log V_{p} & ∼\mathrm{Gamma}(s_{V_{p}},\mu_{V_{p}})\\ T_{p} & ∼\mathrm{lognormal}(\mu_{T},\sigma_{T})\\ \lambda_{g} & ∼\mathrm{Gamma}(s_{\lambda_{g}},\mu_{\lambda_{g}})\\ \;\lambda_{d} & ∼\mathrm{Gamma}(s_{\lambda_{d}},\mu_{\lambda_{d}}), \end{array}\}$$

where *s_i_* and *μ_i_* (*i* = *V_p_*,*λ_g_*,*λ_d_*) are the shape parameter and mean of the gamma distributions, respectively, and *μ_T_* and *σ_T_* are the parameters for the lognormal distribution (mean and standard deviation on the log scale, respectively).

In addition, the viral dynamics described by equation (2.1) were linked to the onset of clinical disease by assuming the time of peak titre and incubation period follow a bivariate lognormal distribution. In this case, the time of onset of clinical signs, *C*, can be written conditionally on the time of peak titre, so that

2.3 $$C|T_{p}∼\mathrm{lognormal}\left(\mu_{C}+\rho_{TC}\sigma_{C}\left(\frac{\log T_{p}-\mu_{T}}{\sigma_{T}}\right),\sigma_{C}\sqrt{1-\rho_{TC}^{2}}\right),$$

where *μ_i_* and *σ_i_* (*i* = *C*,*T*) are the parameters for the (marginal) lognormal distributions (mean and standard deviation on the log scale, respectively) and *ρ_TC_* is the correlation between the times of peak titre and clinical onset. None of the pigs infected with SwIV showed clinical signs [9]. Hence, parameters related to clinical disease (*μ_C_*, *σ_C_* and *ρ_TC_*) were estimated only for FMDV.

### 2.3. Linking within-host dynamics and transmission

Within-host viral dynamics were linked to between-host transmission by assuming that the probability of transmission is related to viral titre. The probability of transmission from an infected to a susceptible animal when exposure occurs between *τ*_0_ and *τ*_1_ days post infection is given by

2.4 $$p=1-\exp\left(-\gamma \int_{\tau_{0}}^{\tau_{1}}S(\tau )\;d\tau \right),$$

where *γ* is the transmission parameter and *S*(*τ*) is the level of viral shedding at *τ* days post infection [20,21]. The transmission parameter captures a range of factors (other than viral load) that influence transmission, including host behaviour and frequency and duration of contacts [22]. Equation (2.4) assumes frequency-dependent transmission, that is the number and rate of contacts for individual is independent of population size [21]. This is often appropriate for directly transmitted viral infections of cattle and pigs, including FMDV [23,24].

Viral shedding was assumed to be proportional to log titre (i.e. *S*(*τ*) = log *V*(*τ*), where *V*(*τ*) is given by equation (2.1)) and the transmission parameter (*γ*) was assumed to be the same for all animals. To test these assumptions, alternative models were considered in which shedding was assumed to be proportional to titre (i.e. *S*(*τ*) = *V*(*τ*)) or in which the transmission parameter varied among animals. When the transmission parameter varied among individuals, the parameter for each animal was drawn from a higher-order distribution. When shedding was proportional to titre, the log-transformed transmission parameters were drawn from a normal distribution (i.e. log *γ_j_* ∼ Normal(*μ_γ_*,*σ_γ_*), where *μ_γ_* and *σ_γ_* are the mean and standard deviation, respectively). When shedding was proportional to log titre, the transmission parameters were drawn from a gamma distribution (i.e. *γ_j_* ∼ Gamma(*s_γ_*,*μ_γ_*), where *s_γ_* and *μ_γ_* are the shape parameter and mean, respectively).

### 2.4. Parameter estimation

Parameters were estimated using Bayesian methods. The likelihood for the data (comprising the virus isolation data, $V_{j}^{\left(\mathrm{obs}\right)}(t_{j})$ measured *t_j_* days post infection, the challenge outcomes, *δ_ij_*, and, for FMDV, the times of clinical onset, *C_j_* for donor animal *j*) is given by

2.5 $$\begin{array}{rl} L(\varphi )= & \prod_{j}(\prod_{t_{j}}\;f\;{\left(\log V_{j}^{\left(\mathrm{obs}\right)}(t_{j})|\log V_{j}(t_{j}),\sigma_{e}^{2}\right)}^{1-d_{t_{j}}}F{\left(0|\log V_{j}(t_{j}),\sigma_{e}^{2}\right)}^{d_{t_{j}}}\\ & \;\times \prod_{i}\;p_{ij}^{\delta_{ij}}{\left(1-p_{ij}\right)}^{1-\delta_{ij}}\\ & \;\times \int_{C_{j}-1}^{C_{j}}g(c)\;dc), \end{array}$$

where **φ** is a vector of model parameters. The first term in the likelihood given by equation (2.5) relates the expected viral titres given by equation (2.1) to the observed titres. Here *f* and *F* are the probability and cumulative density functions for the normal distribution (with mean *V_j_*(*t_j_*) and error standard deviation, *σ_e_*), respectively, and *d* is a variable indicating whether (*d* = 1) or not (*d* = 0) the observation is left-censored (i.e. it is below the detection threshold, set arbitrarily at 1 TCID_50_ ml^−1^ (FMDV) or 1 pfu ml^−1^ (SwIV)). The second term relates to the challenge outcomes for donor *j*, where *p_ij_* is the probability of transmission at the *i*th challenge for the donor (calculated using equation (2.4)). Finally, the third term relates to the onset of clinical disease in donor animal *j*, where *g* is the probability density function for the lognormal distribution conditional on the time of peak titre (given by equation (2.3)). The prior distributions used for each parameter are presented in electronic supplementary material, table S1.

Samples from the joint posterior density were generated using an adaptive Metropolis scheme [25], modified so that the scaling factor was tuned during burn-in to ensure an acceptance rate of between 20% and 40% for more efficient sampling of the target distribution [26]. Two chains of 10 000 000 iterations were run, with the first 5 000 000 iterations discarded to allow burn-in of the chains. Each chain was subsequently thinned by taking every 500th iteration. The adaptive Metropolis scheme was implemented in Matlab (version R2020b; The Mathworks Inc.) and the code is available online [27]. Convergence of the scheme was assessed visually and by examining the Gelman–Rubin statistic in the coda package [28] in R (version 4.0.5) [29].

The four models for viral shedding (i.e. proportional to titre or log titre) and variation in transmission parameters among animals (i.e. common to all animals or varies among animals) were compared using the deviance information criterion (DIC) [30].

For FMDV, the models using viral titres in different compartments (i.e. blood, NF or OPF) were compared by computing posterior predictive *p*-values for transmission by each animal. Specifically, the joint posterior distribution for an animal was sampled and the probability of transmission at each challenge computed. Whether or not transmission occurred at each challenge was then simulated and the observed outcomes of all challenges for the animal were compared with the simulated ones. This was repeated multiple times and the proportion of samples for which the observed and simulated outcomes matched was computed (i.e. the posterior predictive *p*-value).

### 2.5. Summary transmission measures

To explore the relationship between within-host viral dynamics and between-host transmission two measures summarizing transmission between individuals were considered: the reproduction number (*R*); and the generation time (*T_g_*). The reproduction number, *R*, is given by

2.6 $$R=\gamma \int_{0}^{\infty }S(\tau )\;d\tau ,$$

and the generation time, *T_g_*, is given by

2.7 $$T_{g}=\frac{\int_{0}^{\infty }\tau S(\tau )\;d\tau }{\int_{0}^{\infty }S(\tau )\;d\tau },$$

where *γ* is the transmission parameter and *S*(*τ*) is the level of virus shedding by an individual at time *τ* post infection [21,31].

In addition, the proportion of transmission before the onset of clinical signs (denoted by *θ*) was calculated, which is useful for assessing the efficacy of reactive control measures [19,31]. This is given by

2.8 $$\theta =\frac{\int_{0}^{\infty }S(\tau )(1-F(\tau ))\;d\tau }{\int_{0}^{\infty }S(\tau )\;d\tau },$$

where *S*(*τ*) is the level of virus shedding and *F*(*τ*) is the cumulative density function for the incubation period conditional on the time of peak titre (given by equation (2.3)). Because none of the pigs infected with SwIV showed clinical signs, *θ* was only calculated for FMDV in cattle.

## 3. Results

### 3.1. Within-host viral dynamics

**Figure 3.**
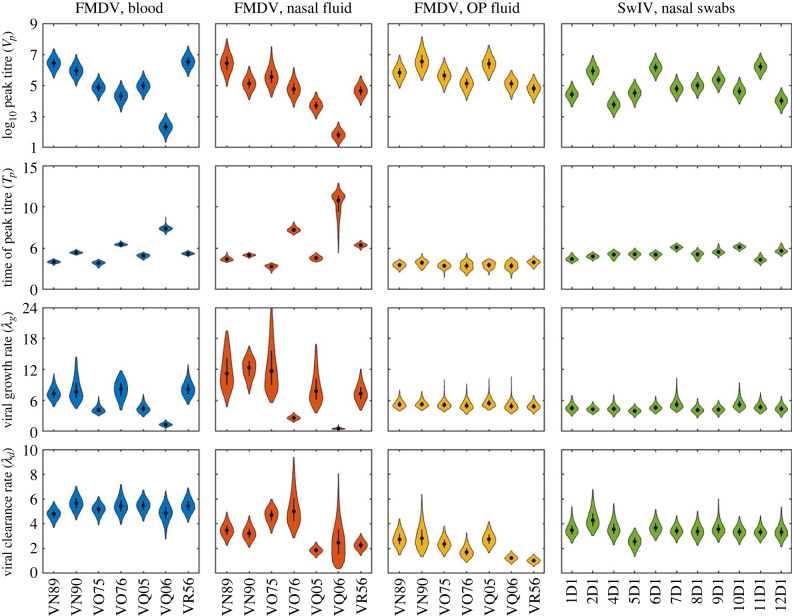
Within-host parameters for cattle infected with foot-and-mouth disease virus (FMDV) and pigs infected with swine influenza virus (SwIV): peak titre (log_10_ *V_p_*; log_10_ tissue culture ID_50_ ml^−1^ for cattle, log_10_ pfu ml^−1^ for pigs); time of peak titre (*T_p_*; days post infection); rate for the exponential viral growth phase (*λ_g_*; per day); and rate for the exponential viral decay phase (*λ_d_*; per day). Violin plots show the posterior density (shape), the posterior median (black circles) and the interquartile range (black line). Shape colour indicates the virus and the viral titre used in the model: FMDV and viral titre in blood (blue), nasal fluid (orange) or oesophageal–pharyngeal (OP) fluid (yellow); or SwIV and viral titre in nasal swabs (green).

Fitted curves of viral titres over time are shown for FMDV in three compartments (blood, NF and OPF) in figure 1 and for SwIV in nasal swabs in figure 2. The estimates for the within-host viral parameters for FMDV and SwIV are presented in figure 3. Estimates for the hierarchical parameters are presented in electronic supplementary material, table S2 for FMDV and in electronic supplementary material, table S3 for SwIV.

The dynamics of FMDV in blood and NF differed among cattle in both peak titre and timing of peak titre, while those in OPF were more consistent in the timing of peak titre (figure 1). This was reflected in differences among animals in all four within-host parameters (*V_p_*, *T_p_*, *λ_g_* and *λ_d_*) for blood and NF, whereas only the peak titre (*V_p_*) in OPF varied greatly among individuals (figure 3). Similarly, the principal difference among pigs in within-host parameters for SwIV was in peak titre, while the remaining parameters were consistent among individuals (figures 2 and 3).

### 3.2. Linking within-host dynamics and transmission

Viral shedding was assumed to be proportional to log titre and the transmission parameter was assumed to be the same for all animals. None of the alternative models in which shedding was assumed to be proportional to titre or in which the transmission parameter varied among animals was better supported by the data for either FMDV or SwIV (electronic supplementary material, table S4). Model fits and parameter estimates for the alternative models are shown and discussed in electronic supplementary material, figures S1–S3 and text S1.

Virus isolation from NF was the best proxy measure for FMDV infectiousness. The model using NF as the proxy adequately captured the challenge outcomes for all seven animals (posterior predictive *p*-values greater than 0.15 for all animals) and had the highest posterior predictive *p*-value in a majority (five out of seven) of comparisons (electronic supplementary material, table S5). By contrast, there were animals for which the models using virus isolation from blood or from OPF as the proxy were not reliably able to capture the challenge outcomes (i.e. posterior predictive *p*-values < 0.05) (electronic supplementary material, table S5). For blood as the proxy this was due to transmission occurring when viral titres were very low, while for OPF as the proxy this was because transmission did not occur when viral titres were high (figure 1).

### 3.3. Relationship between within-host dynamics and transmission

The shedding curve, *S*(*τ*) = log *V*(*τ*), can be approximated by a piecewise linear function (electronic supplementary material, figure S4). This heuristic approximation enables expressions to be derived which explicitly relate the two summary transmission measures, the reproduction number (*R*) (given by equation (2.6)) and the generation time (*T_g_*) (given by equation (2.7)), to the within-host parameters (*V_p_*, *T_p_*, *λ_g_* and *λ_d_*) and transmission parameter (*γ*) (see electronic supplementary material, text S2 for details).

For the reproduction number, this relationship is

3.1 $$R\approx \frac{1}{2}\gamma \left(\frac{1}{\lambda_{g}}+\frac{1}{\lambda_{d}}\right)\log V_{p}(\log V_{p}+\log4),$$

indicating that *R* increases with an increase in transmission parameter (*γ*) or peak viral titre (*V_p_*) and decreases with an increase in rates in the exponential growth or decay phases (*λ_g_* or *λ_d_*), but is independent of the time of peak viraemia (*T_p_*). The corresponding relationship between the generation time and the within-host and transmission parameters is

3.2 $$T_{g}\approx T_{p}+\frac{1}{3}\left(\frac{1}{\lambda_{d}}-\frac{1}{\lambda_{g}}\right)(\log V_{p}+\log2).$$

This shows that *T_g_* increases as the time and level of peak titre (*T_p_* and *V_p_*) and the rate in the exponential growth phase (*λ_g_*) increase, and decreases as the rate in the exponential decay phase (*λ_d_*) increases, but is independent of the transmission parameter (*γ*).

The accuracy of the approximations given by equations (3.1) and (3.2) was checked by comparing the values for *R* and *T_g_* obtained using them with those computed by evaluating equations (2.6) and (2.7) numerically. The median absolute difference for *R* was less than 0.02, while for *T_g_* it was less than 0.005 (electronic supplementary material, table S6). This shows that the approximations are sufficiently accurate for it to be reasonable to draw inferences using them, at least over the range of parameters estimated from the transmission experiments for FMDV and SwIV.

**Figure 4.**
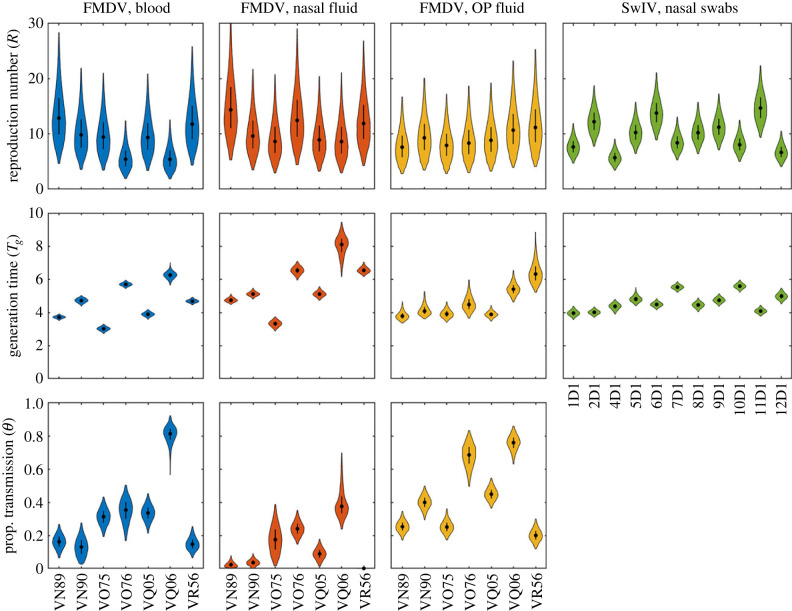
Summary transmission measures for cattle infected with foot-and-mouth disease virus (FMDV) and pigs infected with swine influenza virus (SwIV): reproduction number (*R*); generation time (*T_g_*; days) and proportion of transmission before the onset of clinical signs (*θ*). Violin plots show the posterior density (shape), the posterior median (black circles) and the interquartile range (black line). Shape colour indicates the virus and the viral titre used in the model: FMDV and viral titre in blood (blue), nasal fluid (orange) or oesophageal–pharyngeal (OP) fluid (yellow); or SwIV and viral titre in nasal swabs (green). Because none of the SwIV-infected pigs showed clinical signs, *θ* was not calculated in this case.

**Figure 5.**
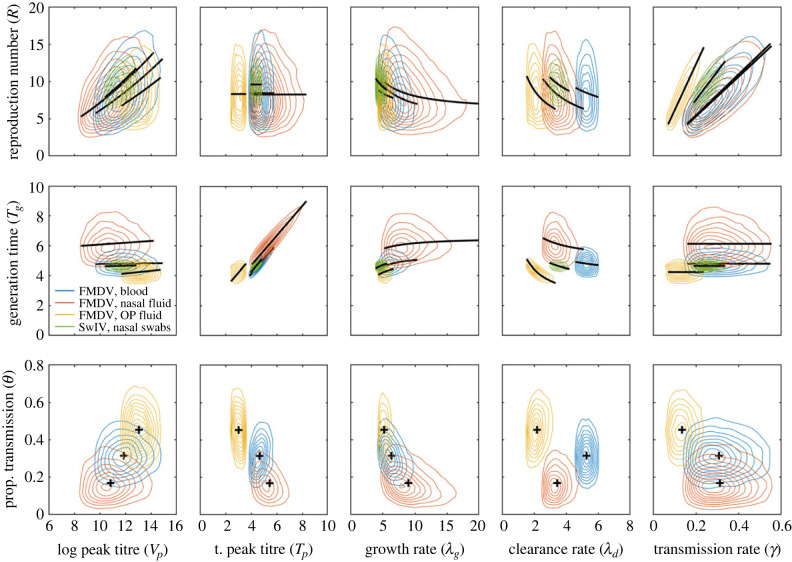
Summary transmission measures for foot-and-mouth disease virus (FMDV) in cattle and swine influenza virus (SwIV) in pigs and their dependence on within-host viral dynamics. Plots show the reproduction number (*R*; top row), generation time (*T_g_*; middle row) or proportion of transmission before the onset of clinical signs (*θ*; bottom row) and their dependence on peak titre (log *V_p_*), the time of peak titre (*T_p_*), the rates during the exponential viral growth (*λ_g_*) and decay (*λ_d_*) phases and the transmission parameter (*γ*). Contours show the joint posterior density for the summary measure and the hierarchical (i.e. population-level) mean for the within-host parameter. Colour indicates the virus and the viral titre used in the model: FMDV and viral titre in blood (blue), nasal fluid (orange) or oesophageal–pharyngeal (OP) fluid (yellow); or SwIV and viral titre in nasal swabs (green). Black lines show the relationship between *R* or *T_g_* and the within-host parameter as defined by equation (3.1) or (3.2), respectively, with the remaining parameters fixed at their posterior median values. Black crosses mark the posterior median for *θ* and the parameter. Because none of the SwIV-infected pigs showed clinical signs, *θ* was not calculated in this case.

Between-animal variation in the within-host parameters (figure 3) resulted in between-animal variation in the reproduction numbers and generation times (figure 4; electronic supplementary material, figures S5 and S6). The relationships between the summary measures and the within-host parameters can be seen when the marginal joint posterior distributions for *R* or *T_g_* and the parameter is compared with the expressions inferred heuristically given by equations (3.1) and (3.2), respectively. This is the case both for the population-level within-host parameters (figure 5) and those for each animal (see electronic supplementary material, figures S5 and S6).

For FMDV, the reproduction number for an animal was broadly similar for each of the proxy measures of infectiousness (figures 4 and 5; electronic supplementary material, figure S5). By contrast, the generation time for an animal was consistently higher when using NF as a proxy compared with blood, but with no clear pattern for OPF (figures 4 and 5; electronic supplementary material, figure S5).

### 3.4. Proportion of transmission before clinical signs

For FMDV, the proportion of transmission before the onset of clinical signs (denoted by *θ*; equation (2.8)) was also calculated for each animal. Again, between-animal variation in within-host dynamics (figure 3) resulted in between-animal variation in *θ* (figure 4; electronic supplementary material, figure S7). The joint posterior distributions for *θ* and each within-host parameter indicate that larger values of *θ* are associated with earlier times of peak titre and lower rates in the exponential growth phase (figure 5; electronic supplementary material, figure S7). Furthermore, the smallest value of *θ* was obtained when NF was used as the proxy measure of infectiousness, followed by blood and then OPF (figures 4 and 5; electronic supplementary material, figure S7).

## 4. Discussion

In this study, the relationship between within-host dynamics and between-host transmission was quantified for two viral diseases of livestock, FMDV in cattle and SwIV in pigs, using empirical data from transmission experiments, thereby allowing underlying assumptions in the modelling approach to be tested. Explicit, if approximate, relationships were derived between the within-host (*T_p_*, log *V_p_*, *λ_g_*, *λ_d_*) and transmission (*γ*) parameters and the reproduction number and generation time (*R* and *T_g_*), given by equations (3.1) and (3.2), respectively.

The four parameters in the model used to describe the within-host dynamics (*T_p_*, log *V_p_*, *λ_g_*, *λ_d_*) each reflect the net effect of a combination of underlying biological processes and so mask some of the inherent complexity of the within-host dynamics. For example, the level and timing of peak viral titre is likely to be related to the innate immune response, in particular the interferon response, while viral clearance relates to the levels of antibody and T cell responses (see [19,32] for FMDV and [33,34] for SwIV). This suggests that *T_p_*, log *V_p_* and *λ_g_* are likely to be influenced primarily by innate immune responses, while *λ_d_* is likely to be influenced primarily by adaptive immune responses.

The choice of modelling approach reflects in part a limitation of using only viral titres when estimating within-host parameters, such that only the four parameters in the model are identifiable from the data [17,35]. The model is nonetheless able to capture the trajectories of the viral titres within a host (figures 1 and 2) and a similar phenomenological approach has been used previously for FMDV [10], influenza virus [17,18] and SARS-CoV-2 [36,37].

More complex models have been developed to describe the within-host dynamics of viruses, including influenza virus [33,38] and FMDV [32,39]. These models incorporate features of the within-host biology, including viral replication in populations of cells and both innate, especially the interferon response, and adaptive immune responses. Parameters in these more complex models can be related to those in the phenomenological virus curve given by equation (2.1) using approximation methods [40,41]. This provides a means of simplifying a more complex within-host model so that it can be embedded in a between-host transmission model [1]. Furthermore, it makes it easier to assess the impact of within-host processes (e.g. viral replication, innate or adaptive immune responses) on between-host transmission in a quantitative manner.

Transmission does not depend solely on the within-host dynamics of a pathogen. Rather it also depends on additional factors, including host behaviour and frequency and duration of contacts, the effects of which are incorporated implicitly in the transmission parameter [22]. The best-supported models for both FMDV and SwIV assumed a common transmission parameter among animals (electronic supplementary material, table S4), implying that between-host variation in these factors is of limited importance in transmission experiments, especially the one-to-one design used in the studies analysed here. This is unlikely to be the case in the field, however, where contacts between animals are likely to be more variable [42–44].

A further question is how the frequency of contacts made by an individual depends on population size [45,46]. In the present study, the contact rate was assumed to be independent of population size (i.e. frequency-dependent transmission). This is appropriate for transmission of, for example, FMDV in a UK cattle farm [24], and makes scaling from transmission experiments to the field more straightforward. However, this may not be the case in other demographic situations, husbandry settings or for other viruses. There is no general means of determining how the frequency of contacts and, hence, the transmission parameter will depend on population size [20,46]. Any dependence of the transmission parameter on population size introduces a corresponding dependence in the reproduction number, but does not affect the relationship between *R* and the within-host parameters (see equation (3.1)). Both the generation time and proportion of transmission before clinical onset are independent of the transmission parameter (see equations (2.7), (2.8) and (3.2)). As a result, neither of these measures is influenced by assumptions about the relationship between transmission and population size.

Although there was no evidence for contact heterogeneity in the transmission experiments, there was still substantial variation among animals in their summary transmission measures (figure 4). This reflects variation among the animals in within-host dynamics for both FMDV and SwIV (figure 3). The level of variation in the parameters differed among viruses and compartments, with the most consistently variable parameter being peak viral titre. Despite this variation, however, all animals had reproduction numbers well above one (figure 4), suggesting that variation at the within-host level may not result in much variation at the population level in this case. This is in contrast with other viruses for which there can be substantial differences among individuals in terms of infectiousness [47]. Such variation can arise through heterogeneities in viral dynamics, as well as in contacts [12,48].

When scaling from within-host dynamics to between-host transmission there are two important issues to consider [1,2]. First, how does viral load relate to shedding and, hence, transmission? Second, what is the most appropriate proxy measure (i.e. viral titre in which compartment) for infectiousness? Model selection suggested that, for FMDV in cattle and SwIV in pigs, models in which viral shedding is proportional to log titre were better supported by the data than ones in which shedding is proportional to titre, particularly if the transmission parameter was common to all animals (electronic supplementary material, table S4). A similar conclusion was also reached for SARS-CoV-2 [11]. Assumptions about shedding have a large impact on estimates for the reproduction number, which can be several orders of magnitude higher (and sometimes unrealistically high) when shedding is proportional to titre compared with when it is proportional to log titre (electronic supplementary material, figure S3).

The analyses for FMDV show that the choice of proxy measure used for infectiousness (i.e. viral titres in different compartments) did not influence model selection (electronic supplementary material, table S4) and, hence, inferences about how transmission relates to viral titre and variation between animals in transmission parameter. However, the choice of proxy measure influenced the estimates of the summary transmission measures: *R*, *T_g_* and *θ* (figures 4 and 5). Consequently, it is essential to determine which, if any, of the proxy measures considered are reliable indicators of infectiousness.

Using NF as a proxy for infectiousness the reproduction number for FMDV in cattle was estimated (posterior median and 95% credible interval) to be 8.2 (2.9, 21.9), with similar estimates for the other proxy measures (figures 4 and 5). This is consistent with estimates published in the literature, which range from 2 to 70 [49–52]. It is, however, lower than the estimate obtained in a previous analysis of the same transmission experiments [19] using challenge outcome only: 22.2 (7.7, 78.1) [49]. The estimate for *θ* using NF as a proxy was 0.18 (0.06, 0.43), which is similar to that reported previously based on the same transmission experiments but analysing challenge outcome only: 0.13 (0.01, 0.44) [19].

The proportion of transmission before the onset of clinical signs, *θ*, can be an indicator of the effectiveness of reactive control measures such as case isolation or, for livestock, reactive culling [19,31]. If a sufficient proportion of transmission is likely to have occurred before clinical signs are detected, reactive control measures would not be expected to be effective (see, for example, fig. 3 in [31]). For FMDV, the estimate for *θ* was 0.18 using NF as the proxy for infectiousness, which suggests reactive control measures are likely to be effective. If, however, blood or OPF is used as the proxy, the estimates for *θ* increase to 0.32 and 0.45, respectively, both of which would indicate reactive control measures are unlikely to be effective. This difference in conclusions emphasizes again the need to determine which proxy measure is a reliable indicator of infectiousness.

## 5. Conclusion

In this study, explicit expressions were derived that relate viral dynamics within a host (level and time of peak titre, virus growth and clearance rates) to between-host transmission (reproduction number and generation time). These expressions were parametrized and the underlying assumptions tested using empirical data from transmission experiments for two viral diseases of livestock, FMDV in cattle and SwIV in pigs. This demonstrated that viral shedding for both viruses is proportional to log viral titre and that, for FMDV, viral titre in nasal fluid is the best proxy for infectiousness. The modelling approach provides a means of embedding more complex within-host models in between-host transmission models. Furthermore, the critical within-host parameters (time and level of peak titre, viral growth and clearance rates) can be computed from more complex within-host models, allowing the impact of within-host processes on between-host transmission to be examined in a more detailed quantitative manner.

## Data Availability

The data used in the study are available from the original publications and are also provided in the electronic supplementary material. All code used in the analyses is available from the Zenodo repository: https://zenodo.org/records/7347011 [27]. Supplementary material is available online [53].
